# Using historical accounts of harpsichord touch to empirically investigate the production and perception of dynamics on the 1788 Taskin

**DOI:** 10.3389/fpsyg.2015.00183

**Published:** 2015-03-11

**Authors:** Jennifer MacRitchie, Giulia Nuti

**Affiliations:** ^1^The MARCS Institute, University of Western SydneySydney, NSW, Australia; ^2^Conservatorio della Svizzera Italiana, University of Applied Sciences and Arts of Southern SwitzerlandLugano, Switzerland

**Keywords:** harpsichord, touch, dynamics, music performance, acoustics, perception

## Abstract

This article investigates the extent of production and perception of dynamic differences on a French historical harpsichord, extensively revised in 1788 by Pascal Taskin. A historical review reports on the descriptions of two different types of touch found in treatises of the 18th century. These two touches (loud/struck and soft/pressed) were used to perform single tones on the lower, upper, *peau de buﬄe* (PDB) registers (the last of which Taskin is credited with having invented) and the coupled 8-foot registers to investigate differences in dynamics. Acoustic measurements show varied differences of up to 11 dB for the two types of touch over different pitches in each register. The strongest difference is measured in the first harmonic of note F2 on the PDB. A listening experiment was conducted to test whether these differences are perceivable. Participants performed a discrimination task using pairs of single tones. Participants were able to perform significantly better than chance in correctly identifying whether pairs of single tones were same or different with respect to loudness [*t*(24) = 12.01, *p* < 0.001]. Accuracies were influenced by pitch and register, the PDB providing the strongest accuracies over the four registers tested.

## INTRODUCTION

When the front of a key on the harpsichord is pressed, the back of the key rises, lifting a jack (a long strip of wood) that holds a piece of quill (the plectrum), which plucks the string. With such a plucking system, dynamic variation has been reported to be mechanically impossible on the harpsichord (Fletcher, 1977), although Benade (1990) writes “The tone color and (to a slight extent) the loudness are both altered when a key is struck more or less hard” (p. 356). Measurements taken on unspecified harpsichords or small plectrum-string models provide much of the evidence for both sides of this argument. However, harpsichords are not a homogenous instrument group and have different characteristics of construction, depending on their provenance and date, which are seldom explored; rarely are the capabilities of historical instruments documented (with the exception of studies by Beurmann and Schneider, 2003). Hall (1993) agrees with Benade (1990) that changes in loudness are possible based on simulations of the plectrum-string interaction and an informal test on an unspecified harpsichord. Griffel’s (1994) model also supports this, claiming that the amplitude of string motion is related to jack velocity, however, Giordano and Winans (1999) refute Griffel’s (1994) claims based on measurements of a small model of the plucking mechanism made with a plastic jack and plectrum. Studies investigating the production and perception of dynamics in harpsichords are rare, with the exception of Penttinen’s (2006) study. Penttinen’s (2006) results show small, measurable and perceivable differences between tones produced with three different striking velocities referred to in the article as pianissimo, mezzo forte, and fortissimo. Although precise details of the different velocity levels or definition of touch used to produce them are not directly stated, the recorded tones are reported to have been achieved on a harpsichord built in 2000 with characteristics adapted from harpsichords built in Italy and southern Germany.

Harpsichords built in France in the second half of the 18th century, such as the instrument considered in this paper, typically have two 8-foot registers (i.e., two complete sets of jacks) which are controlled by the player via two manuals (keyboards). The plucking point of the quill on the string in relation to the bridge differs between registers; the jacks of the upper keyboard are closer to the bridge, while the jacks of the lower keyboard are further away. Depending on the cut of the quill (the voicing of the instrument) the sound of the upper keyboard tends to be softer than the lower keyboard; but most importantly the coupler mechanism on the lower keyboard allows the two 8-foot registers to be played at the same time, and the 4-foot register can also be added (strings at 8-foot pitch sound at normal pitch, strings at 4-foot pitch sound an octave higher). When the sound of the coupled lower keyboard is juxtaposed with the sound of the upper keyboard, dynamic contrast is obtained (when the two 8-foot registers are coupled, two sets of strings are playing, but on the upper keyboard, only one set of strings will play); on French harpsichords dynamic differentiation of large musical sections (rather than individual notes) is therefore successfully accomplished through changes in registration; whereas other expressive means, such as timing, articulation, phrasing, arpeggiation, speed of the spread of a chord and overholding notes are effective in achieving a varied performance for smaller sections of phrases (for instance, changing articulation for individual notes). These techniques are described in French 18th century keyboard treatises (Duphly, 1769 and Foucquet, 1751 among others); they are codified in ornament tables (d’Anglebert, 1689 and Couperin, 1716, revised edition 1717 are two of the most comprehensive tables) and, in some instances, are notated in scores (for example in slow movements by Forqueray (1747) and Duphly (1769)).

It is generally accepted that dynamic contrast on the harpsichord is accomplished through the use of registration and therefore in a different way from the other keyboard instruments in vogue in the late 18th century: the clavichord and piano. These can boast a dynamic range that is obtained by varying the speed with which the key is pressed; the technique of achieving this final abstraction of finger-key contact is referred to as “touch” an umbrella term comprising many aspects such as the finger position, arm joint rigidity, and upper body posture. However, harpsichord treatises from the 17th and 18th centuries do not ignore aspects of performance that have a direct influence on touch, such as how to sit at the instrument, how the arms should be held, how finger movements should be made and, in particular, the lightness with which the keys should be pressed. These are discussed in detail, signifying that touch is considered to be an important element of a harpsichordist’s technique. The centrality of touch in harpsichord performance is also proclaimed by the titles of the published treatises on playing the harpsichord, including those of two of the greatest eighteenth century French harpsichordists: François Couperin’s L’Art de Toucher le Clavecin (1716, revised edition 1717) and Jean-Philippe Rameau’s De la méchanique des doigts sur le clavessin (1724).

Piano touch has been given much attention both theoretically and empirically, with recent studies showing that the position of the fingers (curved versus straight, Parncutt and Troup, 2002) as well as rigidity and the motion of various joints of the arm (Furuya et al., 2010) aid in achieving optimally efficient movements, while the tactile information from the keys is also used to control time-keeping (Goebl and Palmer, 2008). These differences in finger position, type of movement and joint rigidity are discussed in varying degrees amongst current European conservatoire piano teachers (MacRitchie and Zicari, 2012) who use qualitative descriptions such as flexibility and weight, however, these vary in meaning and application amongst teachers. A similar dispute concerning piano touch regards the possibility of producing of varying timbres, the most audible changes between a pressed or struck touch being in the finger-key noise that is produced before the onset of the note (Goebl et al., 2004; Suzuki, 2007) and the presence of key-bottom impact (Goebl et al., 2014). It has been shown that pianists also control varying timbres through touch with a combination of other techniques such as timing, articulation, and pedaling (Bernays and Traube, 2013), with individual differences in the use of these techniques (Bernays and Traube, 2014).

For the harpsichord, however, despite the many descriptions of how to sit at the keyboard and general considerations on how important it is to have a beautiful touch, there is a paucity of historical sources that specifically describe finger movements in relation to sound production, and harpsichord touch has received less modern empirical attention. Recent harpsichord performance studies tend to focus on such aspects as timing, with chosen tempo and, to a lesser extent, note onset asynchrony being indicators of the perceived individual difference between performers (Koren and Gingras, 2014), with individual differences found in the production of key velocity values across performers and between different pieces of repertoire (Gingras et al., 2013). When investigating voice emphasis in MIDI recordings of twelve harpsichordists, Gingras et al. (2009) noted that both detached articulation and increased key velocity were used to emphasize upper voices (although the extent of this key velocity variation between emphasized and un-emphasized voices was deemed modest).

Schools of harpsichord playing today distinguish themselves not only by the way in which registration and performance techniques are used, but also in the ways in which variety of tone can be obtained by changing the position of the hand and the manner in which the fingers strike the keys (much like the distinctions concerning interpretation and technique to be found between French, German and Russian piano schools, seen in Lourenço, 2010). There is, however, no comparable body of literature that describes in detail the finer points of modern schools and approaches to harpsichord technique, performance, and pedagogy, with traditions generally being passed aurally through the master–student paradigm.

In this paper, a historical review provides the motivation for an empirical study. First, we review the few historical treatises from the 18th century that provide physical descriptions of two different ways of striking the key (touches) on the harpsichord, putting forward a hypothesis for the techniques they may be describing. The historical instrument used for the empirical investigation is also described, the 1788 Taskin, chosen because Taskin’s status as one of the greatest French harpsichord builders of his time is undisputed (Hubbard, 1965; Kottick, 2003). The results of an empirical investigation examining the effects of the touches as described in the treatises on this instrument are then presented, addressing the question: can the harpsichord produce measurable differences in loudness through touch? Further to these acoustic measurements, the link between production and perception is investigated by asking: is a listener today able to perceive these dynamic differences?

## HISTORICAL REVIEW

The earliest references specific to touch on a plucked keyboard instrument (harpsichord or spinet) can be found in 1643 (Denis, 1643, 2nd ed. 1650); the last is found in Diderot and d’Alembert’s (1788) edition of the Encyclopédie Méthodique. This review focuses on three sources: Le Gallois (1680), Couperin (1716, revised edition 1717), and Rameau (1724) as they are the only sources to distinctly describe a difference in sound achieved through touch on the harpsichord; Le Gallois (1680) from the point of view of a listener, Couperin (1716, revised edition 1717), and Rameau (1724) as teachers addressing a performer.

### THE HARPSICHORD IN FRANCE

Consistently from the beginning of the 17th century until the close of the 18th century the harpsichord enjoyed a central place in musical tastes in France. In large part royal musical preferences contributed to this centrality. There was a particularly French concentration on “la belle maniere de toucher”; this interest in the diversities of sound that could be produced on the French harpsichord evolved simultaneously with a growing corpus of harpsichord compositions – works written specifically for the harpsichord, not for a generic keyboard instrument as was more the case in Italy and Germany. This research is therefore limited to a consideration of French sources, and considers the properties of a French historical instrument.

### THE TWO MAIN TYPES OF TOUCH DESCRIBED

“There are certainly different ways of playing it [the harpsichord], but these come down in the end to two main styles, to which all the others more or less relate… He [Chambonnières] had both a bright way of playing [*jeu brillant*] and a flowing way of playing [*jeu coulant*], each combined wonderfully well with the other, in such a way that was unsurpassable... his fingers had a delicacy of touch which other players’ fingers did not have. And so whenever he played a chord and then someone else at the same time tried to copy him, playing the same thing, one could nevertheless hear a great difference between the two. The cause of this difference lay in the fact that he had a dexterity, a way of applying his fingers on the keys which was unknown to the others” (Le Gallois, 1680, pp. 68–69). Although this description draws our attention to Chambonnières’ skill in playing a chord, nonetheless Le Gallois (1680) specifies that it was the way he “applied his fingers to the keys” that determined the sound that distinguished him from others. It is not a description of timing between notes, or even arpeggiation, although these may indeed have been a part of the final result. Le Gallois (1680) does not explain exactly how Chambonnières may have physically achieved this variety of touch (the jeu brillant and the contrasting jeu coulant); it is Couperin (1716, revised edition 1717) who describes two types of touch in a direct remark that connects the action of the finger with the sound produced by the instrument: “Delicacy of touch depends also on holding the fingers as close to the keys as possible. It makes sense to believe [apart from (knowing through) experience] that a hand falling from a height, gives a sharper blow than if it strikes from quite near, and that the quill will draw a harder tone from the string” (p. 7). In another didactic study, Rameau (1724), also describes two ways of touching the key, but warns against striking the key with the entire weight of the hand: it is the finger alone that should “fall” [*tombent*] from above, or “flow” [*coulent*] from one key to another: “The fingers must fall onto the keys and not hit them: moreover, they must flow, so to speak, from one to the next in succession; which must serve as a warning regarding the delicacy which you must use when you begin (p. 4). Rousseau (1768) repeats Rameau’s (1724) advice almost word for word showing how, almost half a century later, this element of technique had not been altered. It is interesting to consider what Rameau (1724) might mean by letting the fingers “fall” from above. When read in conjunction with another sentence from the same publication, it becomes clear that movements should be controlled by the joints of the fingers, knuckles and wrist; the weight of the hand or the forearm should not be used: “Never make the touch of your fingers heavy by the effort of your hand. On the contrary, let it be your hand which, by supporting your fingers, makes their touch lighter; this is of great consequence” (p. 4). This emphasizes the lightness with which touch on the harpsichord should be pursued. The care with which Rameau (1724) further describes finger control is remarkable: “see that the finger which releases a key always remains so close to it that it appears to be touching it” (p. 4).

Our interpretation of these passages is to conjecture that Couperin (1716, revised edition 1717) and Rameau (1724) are describing how two different types of sounds can be achieved by using two different touches; a “pressed” touch, where the finger is constantly in contact with the key, both when it is pressed but also during and after its release, and a struck touch where the finger begins at a height and falls at speed onto the key, using the weight of the finger alone. The sources mention that different striking forces will produce a difference in sound, but their definition of this difference is ambiguous. We are hypothesizing that the difference is in dynamic level, or at least dynamic level is a product of the two touches.

### THE INSTRUMENT

Harpsichords are not a uniform instrumental class: this is not just a matter of good and bad instruments but of very differently constructed and purposed instruments. Harpsichords differ in sound attack and decay, registers’ plucking points, materials, size of the keys, amongst other distinguishing features (Hubbard, 1965); what makes them identifiably members of a class is that they are plucked keyboard instruments.

Historical harpsichords give us insights into historical performing techniques; not because the instruments are necessarily of better quality than modern equivalents, but because of their proximity to the composers from the same period whose works we study today. Performing techniques differ between instruments depending on their particular properties; the properties of the instrument influences all associated considerations of sonority, tempi, dynamics and speed of ornamentation, to name an extensive but not exhaustive set of problems in under-determined scores. A historical instrument can help solve many of the interpretative puzzles a performer is faced with when choosing to apply music history to their performance; a performer today playing on historical keyboard instruments is as close as possible to the performance possibilities that would have been available to performers of the time the instrument was built.

Just over a dozen harpsichords that were built or adapted during the reign of Louis XVI survive (Anderson, 2001, 2002); the instrument under consideration in this paper comes from this period. Surviving harpsichords from the 18th century have been analyzed by instrument builders (Bonza, 1997); but their full performance capabilities are inferred rather than accurately portrayed. Such an instrument needs to be brought up to playing condition and then needs to be ‘played in’ to reach its full voice. Access to surviving historical instruments is, however, restricted; it is rare to have the chance to play them, even rarer for non-players to hear them, and rarest of all to gain access to study an instrument sufficiently to allow exploration and recording of its full capacities, indeed to permit the instrument itself to demonstrate its full, working capabilities, and to understand the implications of these in the performance of the music that was written for such instruments.

#### Materials and cut of the plectrum

When considering a historical harpsichord, there are determining limits – the instrument itself and its permanent structures; and there are fluid factors – the parts of the instrument made with perishable materials, which play a major role in the physical, measurable sound production, that are wholly modern, even on a historical instrument.

In our definition, permanent structures of the instrument include: the outside wooden structure of the instrument (the spine, the tail, the bentside and the cheekpiece), the bottom board, the soundboard, internal framing, bridges, the wrest plank, registers, keyboard, wooden parts of the jacks, tuning pins, and hitchpins. The jack rail can define the depth of touch on instruments where it is the only system in place to stop the vertical motion of the jacks. The perishable parts of the instrument include the strings (made of red brass, yellow brass, or iron), the plectrums (made of bird’s quill, or its modern substitute, delrin, or, in the case of the peau de buﬄe (PDB) register, soft leather), the springs on the jacks (made of bristle or thin brass), and the dampers on the jacks (made of cloth). The quill plucks the string and is directly responsible for sound production; the impact of the cut of the quill on touch and, therefore, sound is enormous. In particular, it is the length and the cut of the quill that determine whether the harpsichord has been strongly or limply voiced, defining whether the instrument is loud or quiet, resonant, or weak. The contact time between string and quill is greater with a longer quill, and the thickness of the quill (how much it has been cut underneath) determines the resistance of the quill against the string; the infinite subtleties in the manner of cutting the quill affect the dynamic responsiveness of individual notes. Performers often work closely with the technicians who are voicing the instrument to obtain the desired resistance and cut of the quill, according to personal taste and requirements of the music; the “original” resistance of the quill is impossible to determine. Contemporary evidence merely stresses the importance of a well-voiced instrument: “One must always play very delicately on the keyboard and always have a very well-quilled instrument. I understand that, nevertheless, there are those people who are quite indifferent; perhaps they play equally badly on any instrument at all” (Couperin, 1716, revised edition 1717, p. 45).

In the late 18th century, harpsichord builders experimented with devices which would allow the harpsichord greater expressive capacities: new mechanisms such as knee levers (genouillères) were devised to permit the performer to add and subtract registers while playing, without having to lift the hands from the keyboard. The invention of the PDB stop by Taskin in 1768 enabled a wider dynamic range through the simple change in the material used to pluck the strings (leather instead of quill). Trouflant (in the Encyclopédie Méthodique edition of 1788) writes extensively about the dynamic capabilities the PDB register afforded through the use of touch: “The effect of this leather on the string of the instrument, results in velvety and delicious sounds: one can swell these sounds at will, by pressing more or less strongly on the keyboard; by this means, one can obtain sounds which are full, mellow, sweet, or voluptuous for the most luscious ear. Does one desire sounds that are passionate, soft, dying? The bufle [sic] obeys the touch of the finger, it no longer plucks, but caresses the string; in the end it is touch, the touch alone of the harpsichordist is enough to alternate, without changing either keyboard or registers, these charming vicissitudes” (p. 179). The enthusiasm with which authors reported on Taskin’s invention of the PDB register points to the fact that they were aware of the limited dynamic possibilities available through touch on the other registers and suggests further that instrument builders at the time were interested specifically in increasing the harpsichord’s dynamic possibilities both through the use of registration as well as through touch (Kipnis, 2006; Rowland, 2014). This idea also complies with the increase of complexity and level of detail in the dynamic markings found in keyboard music of the period, which was intended for and played on both harpsichord and fortepiano. This leads our study to empirically investigate the extent of dynamics said to be afforded by the PDB register in comparison with the other registers.

### CONCLUSION OF HISTORICAL REVIEW

An empirical study was designed to investigate whether the historical descriptions of touch could be used to produce dynamic differences specifically on this late 18th century French harpsichord, and in particular on the PDB register, invented specifically to augment the dynamic range of the instrument. In order to produce the two different touches described in the sources cited and discussed above, the following definitions were used:

• “soft touch”: finger resting on the key, depressing the key as slowly as possible, the aim being to allow the plectrum and the string to be in contact for as long as possible.
• “loud touch”: finger strikes the key from above as fast as possible, the aim being that the contact between plectrum and string is reduced to a minimum.

Following from the discussion of the influence of the materials and the cut of the plectrum on the capabilities of an instrument, we acknowledge that changes in string production, aging of the materials within the instrument itself, and replacement materials being of a slightly different constitution than perhaps they were in the 18th century, will all have an impact on the reaction of the harpsichord and the resultant sound, making it impossible to recreate the exact sound the instrument might have had at the time it was built. Our hypotheses remain: these dynamic differences are still possible due to the combination of the performer’s technique and the design of the instrument. For clarity, we report the current condition of the 1788 Taskin as follows:

• the harpsichord is a French, double manual harpsichord built by an anonymous builder in the early 18th century. It was adapted (*ravalé*) by Pascal Taskin in Paris in 1788.
• the instrument was last revoiced by Bonza in 2013. Buzzard feather was used for the two 8-foot registers, as well as the 4-foot register. Leather was used for the PDB register.
• the jacks on the Taskin are not original but are faithful copies of Taskin’s jacks reconstructed in a previous restoration undertaken by Bonza in 2006.
• the registers are original by Taskin.
• the keyboards are not Taskin’s original but a faithful copies reconstructed in a previous restoration undertaken by Barrucchieri in 1980.
• the instrument was last restrung in 1980.
• the jack rail is not original and was rebuilt by Bonza in 2006 as the original has not survived.
• the knee levers are original by Taskin and still in their original state.
• the wrest plank and tuning pins are original by Taskin.
• all other structures are original.

## EMPIRICAL STUDY

This study aims to investigate whether the touches described in the historical sources can be used to produce differences in dynamics on a functioning historical instrument. Does the historical harpsichord as it stands now produce clear acoustic differences in sound, and is a contemporary audience able to perceive the effects? The basic hypothesis was that small dynamic differences would be measurable and perceivable in all registers. An extension of this hypothesis was that we also expected the measurements on the PDB register to produce larger measurable and perceivable differences than the others.

### EXPERIMENT 1: ACOUSTIC STUDY

#### Methods

***Materials and equipment.*** Recordings were made on the Taskin of 1788 by a professional harpsichordist (co-author Giulia Nuti) in February 2013. The harpsichord was tuned by the restorer Augusto Bonza to A4 = 415 Hz using “Kirnberger II” temperament, as it is contemporary with the instrument. The recordings were made via a stereo-pair of microphones placed close to the harpsichord (approximately 1 m).

***Procedure.*** Single tones were played by the same performer with two different types of touch: (1) a loud touch, also defined as a struck touch where the finger approached the key from a height above and (2) a soft touch, also defined as a pressed touch where the finger was resting on the key before the start of the note. These single tones were repeated on four pitches (F2, F3, F4, and F5) and four register combinations: lower, upper, PDB, and the coupled 8-foot registers. Tones were produced successively with alternating touches (four tones for each touch), each time holding the key down for approximately 2.5 s.

***Data analysis.*** From the 128 tones recorded (four each of 4x register, 4x pitch, and 2x touch), 123 were selected on the basis of a clean attack and release (some tones experienced the key sticking at the key-bed or an audible delay between the keypress and the pluck of the plectrum) for the acoustic analysis. A selection of these tones can be found at http://www.artisticresearch.ch/experiment/taskin-samples. To calculate the magnitude spectrum for each tone, it was subjected to a short-time Fourier analysis with window size of 1024 and increment of 512 samples, conducted using the Spectrum function via the libxtract plugin^1^ (Version 0.6.6) for Sonic Visualiser^2^ (Version 2.3). The fundamental frequencies of each pitch were as follows: F2 = 86 Hz, F3 = 172 Hz, F4 = 334.5 Hz, and F5 = 689.1 Hz.

#### Results

In order to confirm that our performer could produce different dynamic levels with the two different touches, we took the Fast Fourier Transform of each recorded signal and calculated the magnitude of the fundamental frequency. The mean magnitude of the fundamental frequency across the set of samples and the SD is reported in **Table 1**. The largest differences between the mean decibel level of loud and soft touches occur on the PDB register with the variability (SD) lowest at the extremes of recorded pitches (F2 and F5). The coupled 8-foot registers show the least convincing differences between the two touches, which may be a result of the mechanism; when the two keyboards are coupled, the finger is only in contact with the lower keyboard 8-foot register – the upper register plays as a result of the coupler but the key is not controlled directly by the finger. From these measurements we predict that the following perceptual test in Experiment 2 should result in higher accuracies in discriminating between the two touches in the extremes of pitches F2 and F5, as well as on the PDB as opposed to the other registers.

**Table 1:** Mean and SD of the magnitude of the fundamental frequency taken across the whole signal for each recorded tone.

		Pitch							
Register	Touch	F2		F3		F4		F5	
		Mean	SD	Mean	SD	Mean	SD	Mean	SD
Lower 8-foot	Loud	-42.13	1.50	-33.60	1.77	-34.20	0.72	-43.45	0.35
	Soft	-45.33	0.24	-35.44	0.28	-36.75	0.45	-47.72	0.92
Upper 8-foot	Loud	-48.89	0.53	-39.15	1.96	-37.32	1.59	-46.18	0.27
	Soft	-52.56	0.86	-42.58	0.24	-41.06	0.06	-48.96	0.28
Coupled	Loud	-44.58	1.42	-34.24	3.06	-38.14	5.27	-44.51	1.52
	Soft	-45.50	0.50	-38.00	3.51	-40.70	3.65	-45.23	1.47
PDB	Loud	-39.98	0.37	-32.11	0.79	-34.32	0.47	-41.26	0.37
	Soft	-48.68	0.35	-36.90	1.68	-37.35	0.39	-45.98	0.14

*All values are reported in decibels (dB).*

In order to compare our acoustic results with other measurements of dynamics in harpsichord playing (Penttinen, 2006), we conducted two similar measures; first we examined the amplitude of the first three harmonics as they evolve over time, and secondly we looked at the relative levels of harmonics.

**FIGURE 1:**
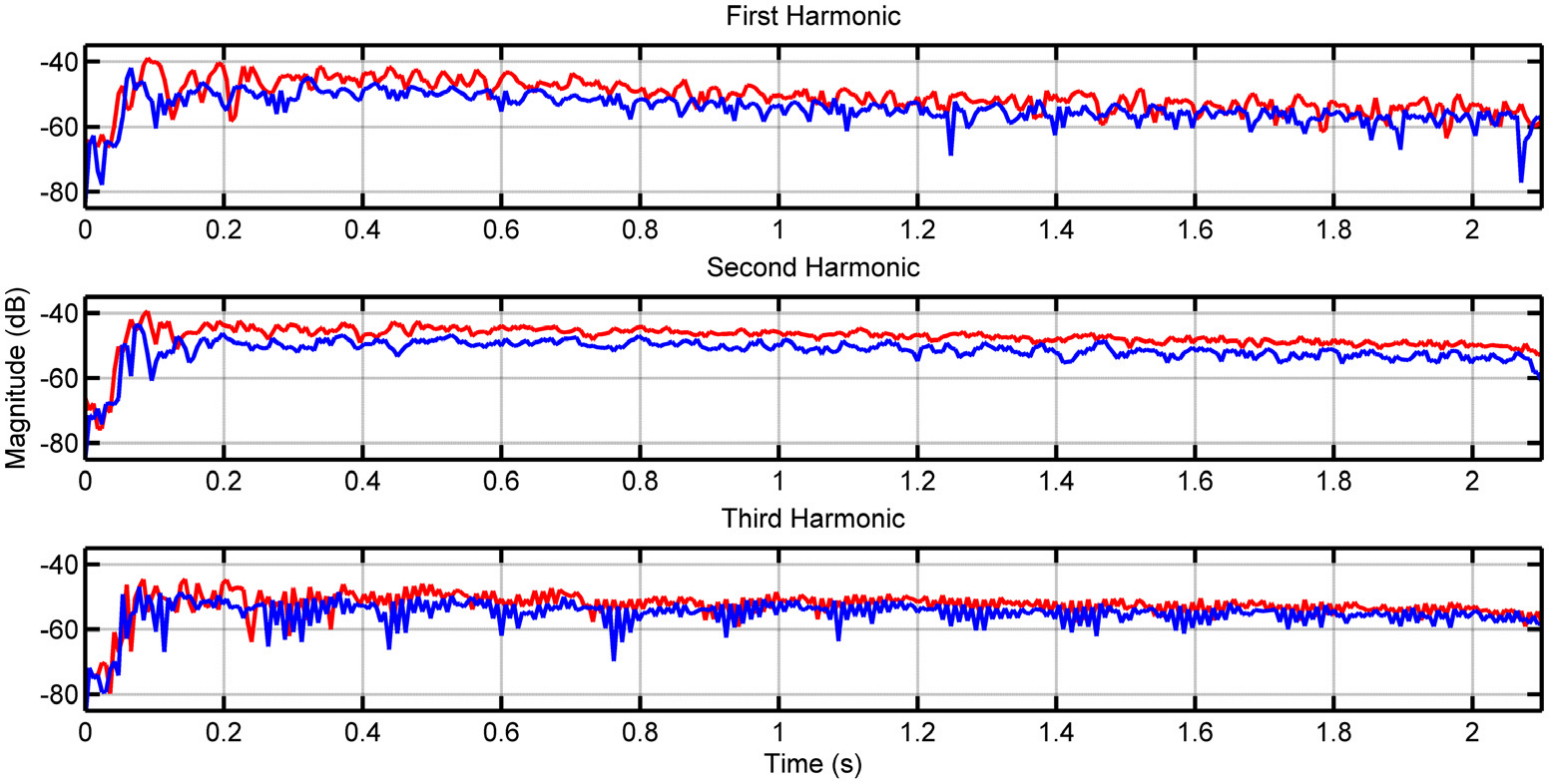
**Envelope of first three harmonics for note F2 played on the lower keyboard.** The loud touch can be seen in red, with the soft touch in blue.

**FIGURE 2:**
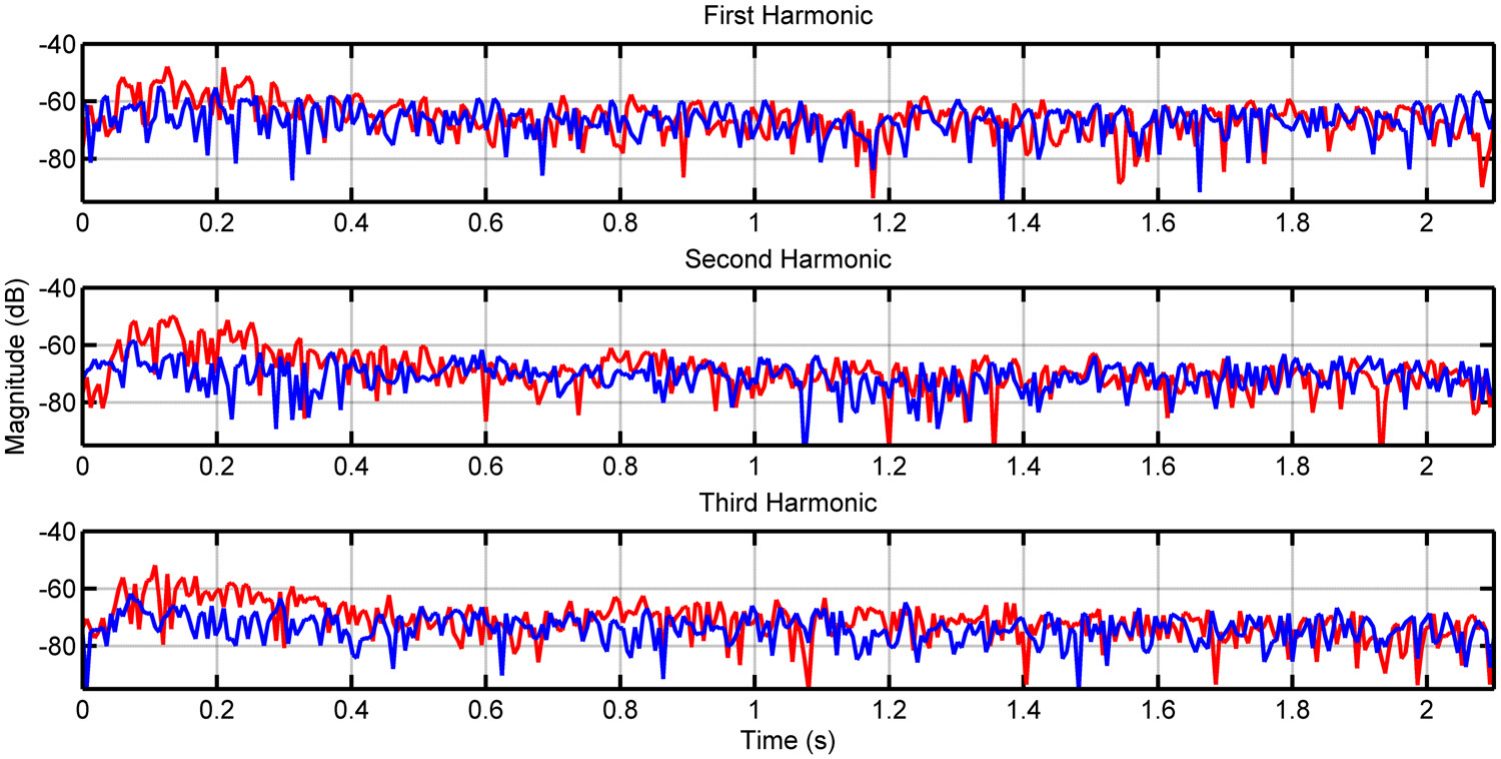
**Envelope of first three harmonics for note F5 played on the lower keyboard.** The loud touch can be seen in red, with the soft touch in blue.

**FIGURE 3:**
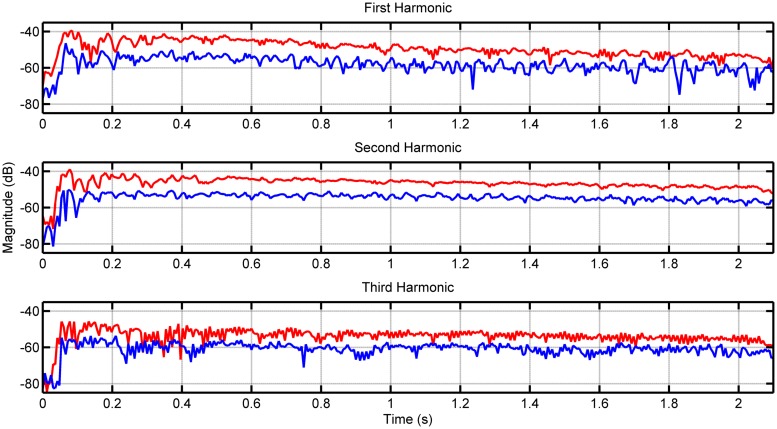
**Envelope of first three harmonics for note F2 played on the *peau de buﬄe* (PDB) register.** The loud touch can be seen in red, with the soft touch in blue.

**FIGURE 4:**
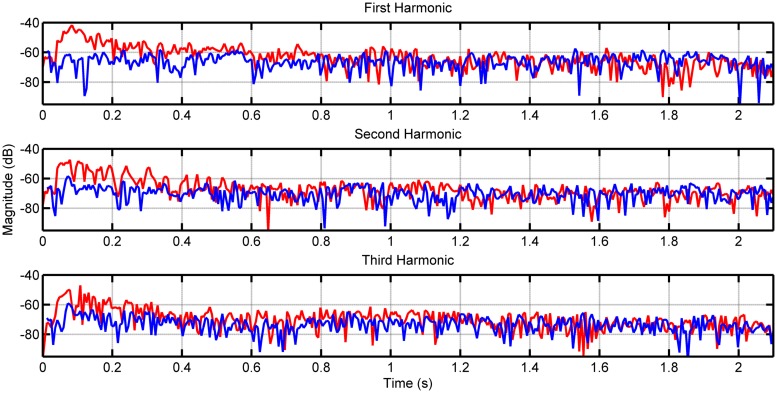
**Envelope of first three harmonics for note F5 played on the PDB register.** The loud touch can be seen in red, with the soft touch in blue.

***Absolute levels:*** **Figure 1** shows the envelopes of the first three harmonics for note F2 on the lower 8-foot register for the first recorded samples of both the loud and soft touches. **Figure 2** shows the envelopes again for the first three harmonics this time for note F5 played on the lower register. As the PDB stop is estimated to produce more audible differences in dynamics, we included similar measurements for notes F2 and F5 in **Figures 3** and **4**, respectively (plots for these pitches in the upper and coupled registers can be seen in the appendices for further comparison).

Although differences in magnitude may be observed in each harmonic between the two types of touch for the lower register, these differences increase visibly when played on the PDB. This is the case particularly for note F2 in **Figure 3**, where the loud touch retains consistently higher amplitude than the soft touch in the first two harmonics. The harmonics of the higher pitches (F5 shown in **Figures 2** and **4**) have noisier envelopes, however, we still observe small differences in the general amplitude of the loud and soft touches. Examining the envelopes of the soft and loud touches, we can see that in general these are similar, however, there are some distinct differences on a more detailed local level (best seen in **Figures 2** and **3**) which are in contrast to the identical harmonic envelopes (albeit with a consistent difference in amplitude) seen in Penttinen (2006). This suggests that the striking velocity could have more of an effect than simply changing the amplitude of string vibration, at least for this particular harpsichord. The absolute levels of amplitude at *t* = 0.4 s are measured for all 123 samples to establish the amplitude at the stable part of the note after the attack (see Weyer, 1976 and Beurmann and Schneider, 2003 for timings of attack transients in harpsichord sounds, seen within the order of 100 ms), and also to provide comparable measurements with Penttinen (2006). **Table 2** displays the measurements for the difference between these amplitude measurements comparing loud and soft touches. The mean and SD of these groups of samples are also included (on average there are four samples for every pitch/register/touch combination). The differences in amplitude between the touches in **Table 2** have a range from -9 dB up to 11 dB (the largest difference is recorded for the PDB register playing note F2). Negative differences are possible for this measurement due to the varying differences in envelope of string vibration between loud and soft touches at any one time instant, as characterized in **Figures 2** and **4**. The largest negative mean differences are located in the recordings of the coupled registers, which also reflect the poor consistency of differences in overall magnitude for this particular combination of registers as seen in **Table 1**. A paired *t*-test on the amplitude values for both touches across register, pitch, and harmonic showed a significant difference of touch [*t*(173) = 7.426, *p* < 0.001] where the loud touches (*M* = -52.986 dB, SD = 13.545 dB) were significantly higher in amplitude than the soft touches (*M* = -56.707 dB, SD = 14.271 dB). This result is interpreted with caution as the groups have been collapsed across frequency and harmonic, which will obviously have an effect on the measured decibel level. However, as most register/pitch/touch combinations only have four samples (some only have three due to spurious sounds in the recordings), further statistical tests cannot be conducted with any strength. A multi-way ANOVA was conducted on the difference in amplitude between loud and soft touches using pitch, register, and harmonics as factors. No effects were found.

**Table 2:** Differences in magnitude between loud and soft touches for each register, pitch, and harmonic combination (value taken at *t* = 0.4s).

Register	Pitch	Harmonic	Difference (dBs) between mean loud and mean soft	Mean loud (dB)	SD loud (dB)	Mean soft (dB)	SD soft (dB)
Lower	F2	1	2.83	-49.08	2.92	-51.90	0.86
Lower	F2	2	0.97	-48.92	3.20	-49.89	0.94
Lower	F2	3	2.24	-51.73	1.63	-53.98	3.06

Lower	F3	1	2.20	-35.51	1.61	-37.70	0.37
Lower	F3	2	3.79	-45.30	0.46	-49.10	0.54
Lower	F3	3	3.86	-47.55	1.56	-51.41	0.96

Lower	F4	1	2.55	-36.86	0.87	-39.40	0.67
Lower	F4	2	3.01	-48.37	0.73	-51.38	0.80
Lower	F4	3	-0.12	-62.50	3.05	-62.38	1.65

Lower	F5	1	2.18	-52.60	0.21	-54.78	1.84
Lower	F5	2	6.51	-63.75	1.25	-70.26	4.93
Lower	F5	3	9.53	-84.41	0.38	-93.94	7.84


Upper	F2	1	-2.20	-58.91	7.08	-56.71	4.76
Upper	F2	2	5.83	-53.52	3.09	-59.35	4.10
Upper	F2	3	1.54	-49.52	5.08	-51.06	1.02

Upper	F3	1	3.13	-41.94	2.94	-45.07	0.60
Upper	F3	2	7.69	-42.73	0.76	-50.42	0.51
Upper	F3	3	5.42	-49.25	1.82	-54.66	1.29

Upper	F4	1	3.95	-40.28	1.83	-44.23	0.24
Upper	F4	2	5.72	-49.12	1.68	-54.85	1.74
Upper	F4	3	2.19	-62.45	3.94	-64.64	2.38

Upper	F5	1	2.93	-48.93	0.49	-51.86	0.43
Upper	F5	2	-0.18	-61.87	2.15	-61.69	0.72
Upper	F5	3	10.43	-80.48	0.88	-90.91	6.17


Coupled	F2	1	-9.39	-60.51	3.81	-51.12	4.12
Coupled	F2	2	6.93	-45.22	1.33	-52.15	2.72
Coupled	F2	3	3.81	-49.00	4.65	-52.81	0.99

Coupled	F3	1	5.19	-34.65	2.89	-39.84	4.08
Coupled	F3	2	2.18	-44.15	1.74	-46.33	0.93
Coupled	F3	3	8.47	-45.23	3.20	-53.69	3.21

Coupled	F4	1	5.89	-41.29	5.41	-47.18	8.16
Coupled	F4	2	4.67	-46.67	2.43	-51.34	7.76
Coupled	F4	3	1.08	-59.62	1.76	-60.69	4.45

Coupled	F5	1	-8.79	-56.00	14.22	-47.21	0.85
Coupled	F5	2	3.67	-58.49	2.83	-62.16	3.17
Coupled	F5	3	5.18	-78.04	2.01	-83.23	0.80


PDB	F2	1	11.29	-44.40	1.38	-55.70	1.41
PDB	F2	2	5.64	-47.10	2.91	-52.74	1.04
PDB	F2	3	0.21	-59.21	6.52	-59.42	1.44

PDB	F3	1	5.64	-33.99	0.49	-39.63	3.04
PDB	F3	2	6.03	-47.82	0.76	-53.85	4.08
PDB	F3	3	10.97	-47.15	0.70	-58.11	2.81

PDB	F4	1	3.30	-36.92	0.62	-40.22	0.49
PDB	F4	2	2.45	-47.82	1.34	-50.27	0.53
PDB	F4	3	10.14	-62.57	2.73	-72.71	6.36

PDB	F5	1	-0.36	-53.53	0.93	-53.17	0.31
PDB	F5	2	5.86	-67.95	1.78	-73.80	2.18
PDB	F5	3	4.54	-97.16	12.01	-101.70	12.53

**FIGURE 5:**
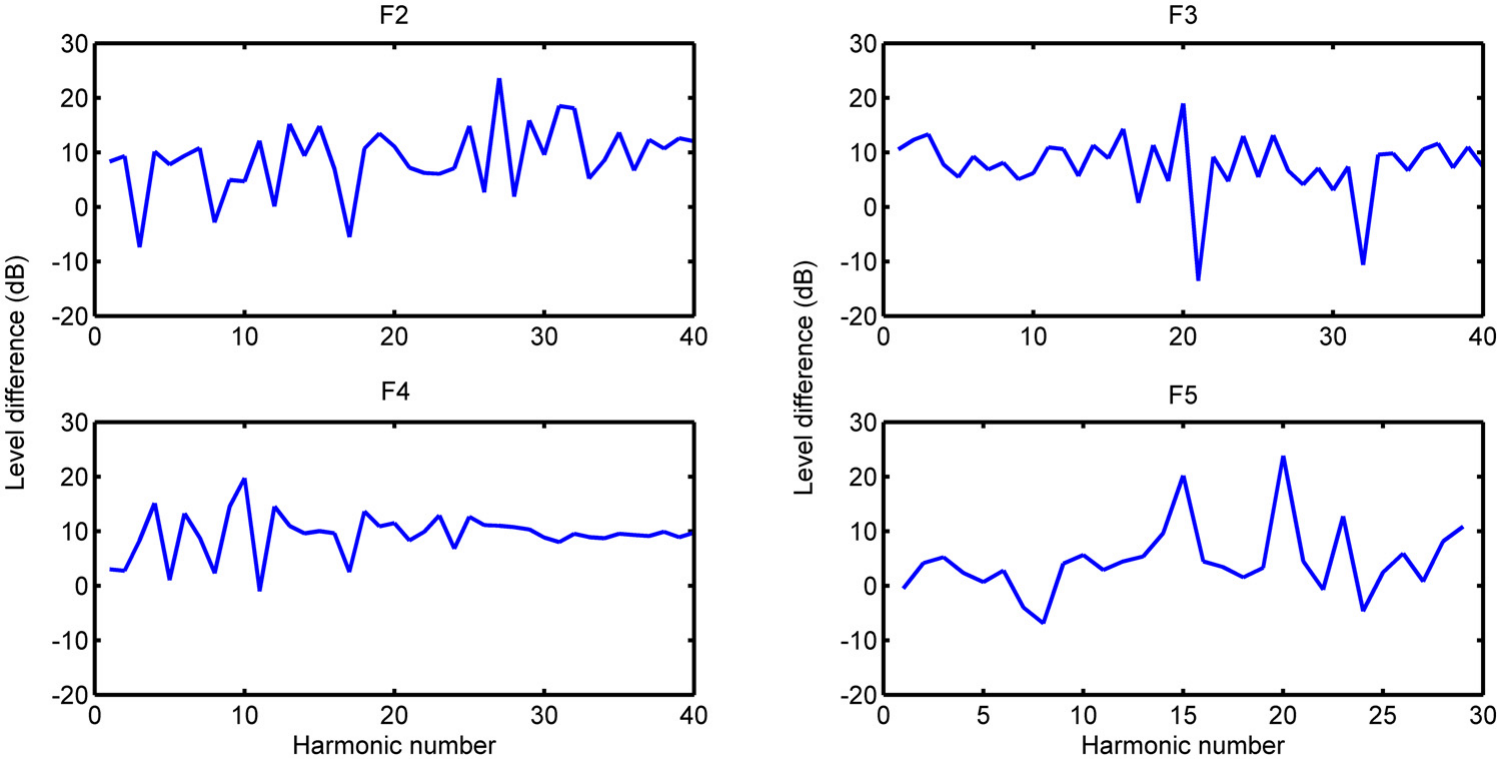
**Relative levels of harmonics (PDB)**.

**FIGURE 6:**
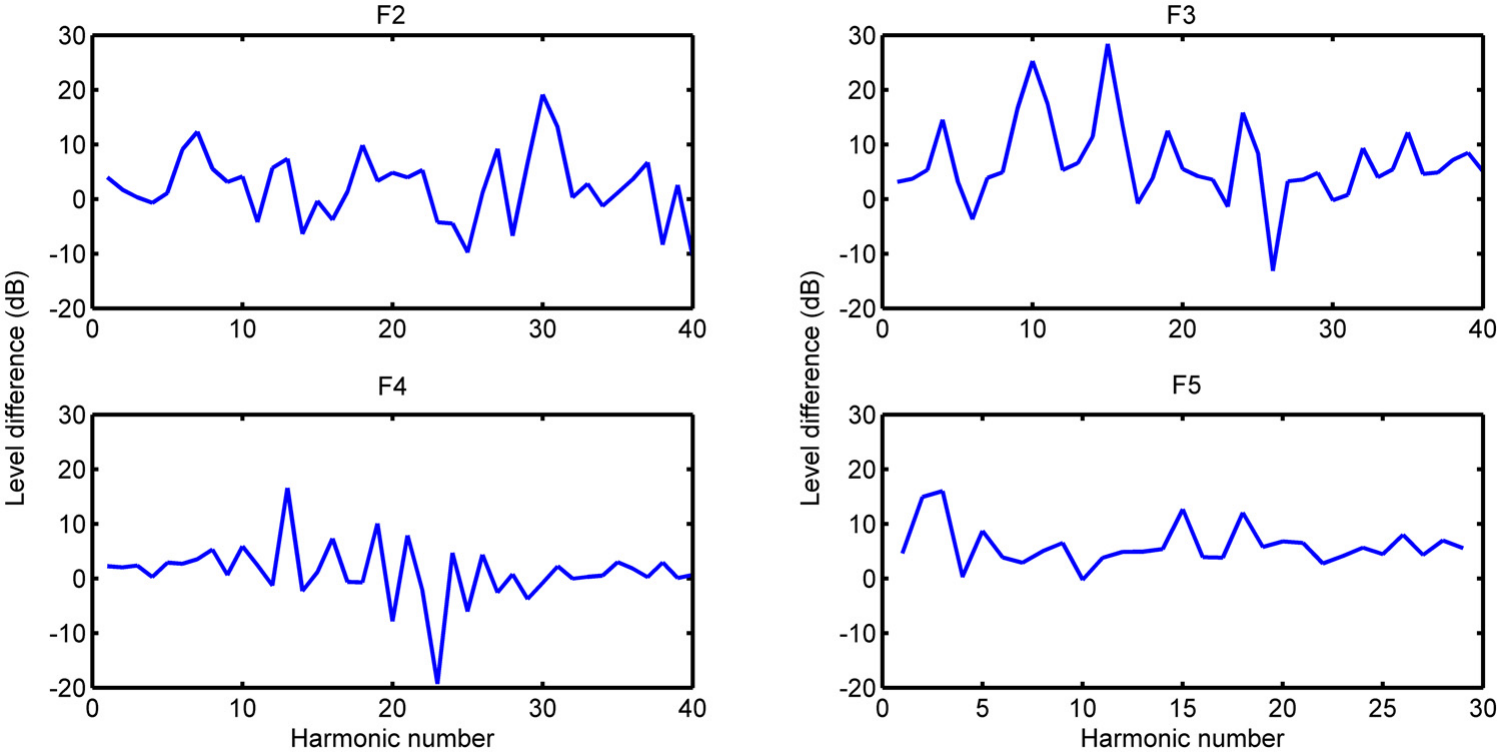
**Relative levels of harmonics (lower)**.

***Relative levels.*** These relative levels are calculated from the difference between the soft and loud touches again at *t* = 0.4 s, allowing us to compare the magnitude of difference in decibels as a function of harmonic index. From the relative levels of harmonics for the lower 8-foot and PDB registers (seen in **Figures 5** and **6**, respectively), there is no general increase in difference that would be expected as a function of excitation force (this result is also confirmed for the harpsichord used in Penttinen, 2006).

### EXPERIMENT 2: PERCEPTUAL STUDY

#### Methods

***Stimuli.*** A selection of single tones recorded in Experiment 1 was used as stimuli. From a set of 123 recorded tones, 64 were selected such that there were two sound examples for each type of stimulus (4x register, 4x pitch, 2x touch). The first two tones in the set of recordings were selected with no other specific criteria applied. Each recorded tone was cut so that there was 50 ms of silence before the attack, and the note itself had a duration of approximately 2 s. A pilot study presenting pairs of these single tones to participants suggested that the finger-key contact noise (or possibly the mechanical noise of the jack hitting the jack rail) may be used to identify the loud or soft touch. The key mechanism sound for this particular harpsichord was recorded in isolation by moving the 8-foot lower register so the plectrum did not touch the strings as the jack traveled upwards. Key F3 was used to produce the ‘knock’. Analyzing the spectrum of this mechanical sound, a loud touch had a peak magnitude of -42.5 dB at the peak frequency of 172 Hz and a soft touch had a peak magnitude of -42.0 dB at the same peak frequency. The loud touch mechanical sound showed higher amplitudes at frequencies above 9 kHz compared to the soft touch suggesting that there is a change in the spectrum of the knock produced depending on the type of touch. To create a set of truncated stimuli without these knocking sounds, we selected two pitches from each register from the original set of 64 stimuli, based on those sounds which showed the highest rate of correct identification in the pilot study, and removed the first 250 ms of the sound. After 250 ms in each isolated knocking sound there is a 10 dB decrease in both recordings (a 10 dB decrease meaning the sound is now a 10th of its original magnitude). This truncation of the first 250 ms removes the initial part of the sound such that the four phases of attack transients, as detailed in Beurmann and Schneider (2003), were absent. Sets of four tones were created which were all equal in pitch, register, and length (i.e., the set were either of original tones, or truncated tones), two tones were performed with a loud touch and two tones were performed with a soft touch.

***Participants.*** Twenty-five participants (10 male, 15 female, age range = 19–33) were recruited from the Masters and Bachelor of Music programmes at the Conservatorio della Svizzera Italiana. None of these participants played the harpsichord. Ethics were followed in participant data collection as set out by the guidelines produced by the British Psychological Society. Participants gave written consent and were advised they could abort the experiment at any time, discarding their data.

***Apparatus.*** Participants were presented these tones in individual sessions through the Presentation software^3^. Listening through Roland RH-5 headphones with a controlled volume level, the participants entered their judgments on the computer keyboard when prompted.

***Procedure.*** Participants were presented pairs of these single tones, both equal in pitch, register, and duration (i.e., whole or truncated) in a 3-alternative forced choice paradigm. The pairs were randomized in terms of pitch, register, and duration as well as the presentation order of each pair (i.e., two loud tones, two soft tones, a loud tone then a soft tone or vice versa), such that all possible comparisons within each group of samples (*n* = 6) were presented. Participants were asked if they could hear a difference in loudness between two sounds, and presented a choice of three answers: (1) sound A was louder than sound B, (2) sound B was louder than sound A, and (3) both sounds were of equal loudness. Participants were allowed to listen again to the pair of sounds for up to two more times to make sure of their judgment. A small set of practice trials preceded the main experimental block of stimuli, using other recorded tones outside the sample set for this experiment.

#### Results

This test was designed to measure whether participants could discriminate between single tones produced with ‘soft’ and ‘loud’ touches on the harpsichord, with respect to loudness. The results of this discrimination task were analyzed using signal detection theory, and as such, participants’ answers have been collapsed into either ‘same’ or ‘different’ responses. Accuracy has been calculated in the form of a *d*-prime (*d*′): *d*′ = *z*(H)-*z*(F), where H is the hit rate and F is the false alarm rate. Hit rate is the number of correct ‘different’ responses to the pairs of tones that were different (in either loud first or soft first presentation orders) divided by the number of ‘different’ trials. False alarm rate is the number of incorrect ‘different’ responses when the stimulus tones were actually of equal loudness, divided by the number of ‘same’ trials. A *d*′ score of 0 reflects chance level responding.

Over all pitches and registers, participants were able to correctly discriminate whether the two tones presented were same or different, performing significantly better than chance level [*t*(24) = 12.01, *p* < 0.001]. This upholds our main hypothesis that participants can perceive changes in dynamics between the two different types of touch. Although some participants informally reported that they attended to the attack of each note in order to make their judgment, in the truncated notes comparison, participants still performed significantly better than chance level [*t*(24) = 10.453, *p* < 0.001]. There was no significant difference between the accuracy rates for the full notes (*M* = 1.588, SD = 0.661) and the truncated notes (*M* = 1.321, SD = 0.632), suggesting that participants are still able to discriminate between soft and loud touches without attack information present in the tone.

**FIGURE 7:**
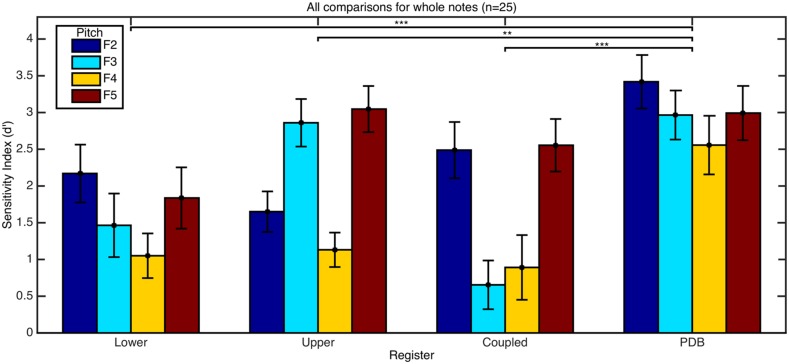
**Mean *d*-prime score as a function of register and pitch for comparisons of whole tones.** Error bars refer to the SE of the mean. Asterisks denote significant differences (**p < 0.01, ***p < 0.001) between mean *d*′ scores of groups by register which are detailed in **Table 3**.

**FIGURE 8:**
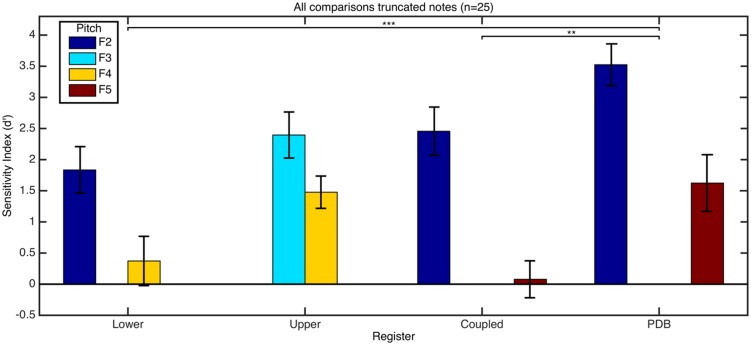
**Mean *d*-prime score as a function of register and pitch for comparisons of truncated tones. Error bars refer to the SE of the mean.** Asterisks denote the significantly higher accuracy in responses to the PDB register (**p < 0.01, ***p < 0.001) when compared to responses to the lower and coupled registers.

From the signal analysis in Experiment 1 we hypothesised that responses to the PDB register would show higher accuracies than the other registers, and that responses to pitches F2 and F5 would be more accurate than to pitches F3 and F4. The response accuracies for each register and each pitch are presented for the blocks with whole notes (**Figure 7**) and truncated notes (**Figure 8**). A two-way ANOVA showed a significant interaction on pitch and register [*F*(9,384) = 2.86, *p* = 0.003] for the whole notes. **Table 3** shows the means and SD for each pitch and register group. Using *post hoc* bonferroni corrected *t*-tests to confirm our original hypotheses, results showed that responses to the PDB were significantly stronger than the other registers [PDB and lower register: *t*(99) = 5.033, *p* < 0.001, PDB and upper register: *t*(99) = 3.299, *p* = 0.001, PDB and coupled register: *t*(99) = 4.832, *p* < 0.001]. Our hypothesis held that responses to pitch F4 would be significantly less accurate than the responses to pitches F2 [*t*(99) = 3.874, *p* < 0.001] and F5 [*t*(99) = 4.544, *p* < 0.001], however, responses to pitch F3 compared to responses to pitch F5 were not significant after bonferroni correction. The responses to the upper register are different to that of the other registers, where discrimination between recordings of pitch F3 is more accurate than that of pitch F2. From the reported mean amplitude levels of the fundamental frequency for each touch in **Table 1**, it is surprising that participants are still able to accurately discriminate between the loud and soft touches in the coupled register, however, the differences in string vibration envelope may contribute to this. A chi-squared test on the number of correct answers for the presentation order of each pair of sounds was found [χ^2^(2) = 32.0, *p* < 0.001] with a soft touch presented before a loud touch being rated more accurately (ratio of correct answers to number of trials = 0.810) than two touches of equal loudness (0.717) or a loud touch followed by a soft touch (0.695). This suggests that an increase in loudness is easier to identify than a decrease (see Olsen, 2014 for a discussion on forward masking of intensity in loudness perception, and Olsen et al., 2010 concerning the effect of recency).

**Table 3:** Group mean and SD of *d*-prime values shown in **Figure 7** listed by register and pitch.

Register	*d*′ values		Pitch	*d*′ values	
	Mean	SD		Mean	SD
Lower	1.631	1.965	F2	2.432	1.872
Upper	2.172	1.638	F3	1.986	2.016
Coupled	1.647	2.070	F4	1.408	1.867
PDB	2.983	1.834	F5	2.608	1.868

For the truncated tones, all accuracies remain significantly better than chance level with the exceptions of the F5 note on the coupled register and the F4 note on the lower register. A two-way ANOVA on pitch (low vs. high) and register showed no significant interaction. A significant main effect of pitch was found [*F*(1,192) = 41.83, *p* < 0.001] with significantly better accuracy in response to lower pitches (*M* = 2.552, SD = 1.911) than higher pitches (*M* = 0.888, SD = 1.899). A significant main effect of register was also found [*F*(3,192) = 6.85, *p* < 0.001] with *post hoc* bonferroni corrected *t*-tests confirming responses to the PDB (*M* = 2.574, SD = 2.200) were significantly higher than responses to the lower register [*M* = 1.103, SD = 2.045, *t*(49) = 3.463, *p* < 0.001] and to the coupled register [*M* = 1.267, SD = 2.090, *t*(49) = 3.046, *p* = 0.003]. A chi-squared test found an effect of presentation order of the two samples [χ2(2) = 15.7, *p* < 0.001] with participants being more accurate in identifying touches of equal loudness (ratio of correct answers to number of trials = 0.742) than decreasing (0.639) or increasing loudness (0.618). This suggests that when the attack information is not present, it is easier to identify equal loudness than any increase or decrease.

## DISCUSSION

The empirical part of this study looked at the production and perception of two types of touch (loud/struck and soft/pressed). From the acoustic results we see that there are clear differences between two types of touch in all registers, however, these are measurably larger in the PDB register, which confirms our original hypotheses. These measured differences vary within each register in terms of pitch, although no distinct pattern is visible in terms of harmonic or pitch over each register. The perceptual results instead suggest that the highest and lowest octaves (in our case octaves 2 and 5) produce the highest accuracies when identifying differences between the two touches within each register, with accuracies in responses to the tones played on the PDB significantly higher than the other registers. Perceiving touch differences in higher octaves may also benefit from the mechanical noise present in the attack of the note. These small registered differences in dynamics may aid the performer to distinguish different voices, particularly once moved away from the central octaves. Although this result is for the Taskin, this may suggest why Gingras et al. (2009) noted on their MIDI-equipped harpsichord that performers used increased key velocities when emphasizing upper voices. In terms of larger dynamic differences, it is true that even though the Taskin would not be capable of the range of dynamics seen on a modern-day piano, measured differences of up to 11 dB in the PDB register for the first harmonic represent a sizeable difference in amplitude. For a performer, full appreciation of the possibility of achieving varied sounds, not just through articulation but also through the type of touch used, raises awareness of what can be achieved in terms of dynamics, inspiring a broader investigation of the technical skills that can be used on the harpsichord, together with a more careful consideration of their effect.

Although we have measured just one historical instrument, it would be interesting to compare dynamic capabilities of other instruments by Taskin, and also extend this type of study to other styles of harpsichords (French, German, Italian etc.). Considering differences in recording setup and venue, we cannot make a direct comparison concerning absolute values of amplitude to the study by Penttinen (2006), however, this study contributes another set of measurements on a different type of harpsichord to the discussion, showing that far from being completely negligible, there are indeed measurable and perceptible (although limited) differences in dynamics. In this study, a single performer was successful in achieving measurable and perceptible dynamic differences, although as seen in results from Gingras et al. (2013), individual differences could play a part in the production and perception of these notes. The varied methods of touch in different schools of harpsichord playing may provide different results and warrant further investigation.

Further investigation is necessary to place these results in a musical context: although our performer made a measurable and perceivable distinction between a loud and a soft touch in consecutive single notes, would this be possible to the same extent within complex musical passages where several voices may be required from the same hand? It is suggested that creating overall dynamic differences in the harpsichord may be akin to creating timbral differences in the piano (as seen in Bernays and Traube, 2013): it may not be one sole technique that is the contributing factor to dynamics perception but rather the combination of techniques such as timing, articulation, and (in obviously a limited extent compared to the piano) differences in the dynamics of each note which can be achieved through touch.

## Conflict of Interest Statement

The authors declare that the research was conducted in the absence of any commercial or financial relationships that could be construed as a potential conflict of interest.

## Footnotes

1 http://jamiebullock.github.io/LibXtract

2 http://www.sonicvisualiser.org

3 http://www.neurobs.com/presentation
