# Supplementary material for: Using historical accounts of harpsichord touch to empirically investigate the production and perception of dynamics on the 1788 Taskin
Source: Front Psychol. 2015 Mar 11;6:183. doi: 10.3389/fpsyg.2015.00183 (PMC4366653; doi:10.3389/fpsyg.2015.00183)
Supplement: Supplementary file 1 [file data_sheet_2.docx]

**Appendix 1: French quotations (translated by co-author Giulia Nuti)**

**Le Gallois (1680), *Lettre de Mr Le Gallois, à Mademoiselle Solier touchant la musique* (p.68-69, complete passage), quoted lines128 - 135:**

Et c’est ce que nous ne pouvons mieux confirmer que par l’exemple du Clavessin; parce que c’est l’instrument de tous le plus parfait. Il est certain qu’il y a diverses manières d’en joüer, qui se réduisent à deux principales, dont les autres participent plus ou moins: Et comme chaque Maître affecte quelqu’une de ces manieres, cela fait que chacun de leurs écoliers prevenu de la maniere de joüer de son Maître, blâme velle des autres tres-souvent sans raison, faute de s’y connoître.

La premiere est cette belle & agreable maniere dont few Chambonniere se servoit. Tout le monde sçait que cet illustre personnage a excellé par dessus les autres, tant à cause des pieces qu’il a composées, que parce qu’il a esté la source de la belle maniere de toucher, où il fasoit paroître un jeu brillant & un jeu coulant si bien conduit & si bien ménagé l’un avec l’autre qu’il estoit impossible de mieux faire. On sçait qu’outre la science & la netteté, il avoit une delicatesse de main que les autres n’avoient pas; de sorte que s’il faisant la même chose, on y trouvoit neanmoins une grande difference; & la raison est, coome j’ay dit, qu’il avoit une adresse & une maniere d’appliquer les doigts sur les touches qui estoit inconnuë aux autres.

**Couperin (1717), *L’Art de Toucher le Clavecin* (p. 7), quoted lines 143 - 146:**

La douceur du toucher dépend encore de tenir ses doigts le plus prés des touches qu’il est possible. Il est sensé de croire (l’experience àpart), qu’une main qui tombe de hault donne un coup plus sec, que sy elle touchoit de prés; et que la plume tire un son plus dur de la corde.

**Rameau (1724), “De la mechanique des doigts sur le clavessin”, in *Pieces de clavecin avec une table pour les agremens* (p. 4), quoted lines 150 - 153:**

Il faut que les doigts tombent sur les touches, & non pas qu’il les frappent; il faut de plus qu’il coulent, pour ainsi dire, de l’un à l’autre en se succedant: ce qui doit vous prevenir sur la douceur avec laquelle vous devez vous y prendre en commencant.

**Rameau (1724), “De la mechanique des doigts sur le clavessin”, in Pieces de clavecin avec une table pour les agremens (p. 4), quoted lines 158 - 160:**

La jointure du poignet doit toujours être souple: cette souplesse qui se répand pour lors sur les doigts, leur donne toute la liberté & toute la legereté nécessaires.

**Couperin (1717), *L’Art de Toucher le Clavecin* (p. 5), quoted lines 165 - 168:**

Sy une personne a un poignet trop hault en jouant, le seul remède que j’aye trouvé, est de faire tenir une petitte baguétte-pliante par quelqu’un; laquelle sera passée par dessous L’autre poignet. Sy le déffaut est opposé, on fera le contraire.

**Rameau (1724), “De la mechanique des doigts sur le clavessin”, in *Pieces de clavecin avec une table pour les agremens*(p. 4), quoted lines 170 - 172:**

Le mouvement des doigts se prend à leur racine, c’est-à-dire, à la jointure qui les attache à la main, & jamais ailleurs; celui de la main, se prend à la jointure du poignet, & celui du bras, supposé qu’il soit necessaire, se prend à la jointure du coude.

**Rameau (1724), “De la mechanique des doigts sur le clavessin”, in *Pieces de clavecin avec une table pour les agremens*(p. 4), quoted lines 174 - 176:**

N’appesentissez jamais le toucher de vos doigts par l’effort de votre main; que ce soit au contraire votre main qui en souvenant vos doigts, rende leur toucher plus leger: cela est d’une grande conséquense.

**Rameau (1724), “De la mechanique des doigts sur le clavessin”, in *Pieces de clavecin avec une table pour les agremens*(p. 4), quoted lines 178 - 179:**

Observez que le doigt qui quitte une touche, en soit toujours si proche, qu’il paroisse la toucher.

**Couperin (1717), *L’Art de Toucher le Clavecin* (p. 45), quoted lines 213 - 215:**

Il faut surtout se rendre tres dèlicat en claviers; et avoir toujours un instrument bien emplumé. Je comprens cependant qu’il y à des gens a qui cela peut estre indiffèrent; parcequ’ils joüent ègalement mal sur quelqu’instrument que ce soit.

**Canon Trouflant, in Diderot, D. and d’Alembert, J. (eds.) (1788). *Encyclopedédie méthodique. Arts et metiers mecaniques*. Paris: Panckoucke, Liège, Pomteux (p.179), quoted lines 222 – 228:**

D’esset de cette peau sur la corde de l’instrument, il résulte des sons voloutés & délicieux: on enfle ces sons à volonté, en appuyant plus ou moins fort sur le clavier; par ce moyen, on obtinent des sons nourris, moëlleux, suaves, ou plutôt voluptueux pour l’oreille la plus épicurienne. Desire-t-on des sons passionés, tendres, mourans? Le bufle obéit à l’impression du doigt, il ne pince plus, mais il caresse de la corde; le tact enfin, le tact seul du claveciniste suffit pour opérer alternativement, & sans changer ni de clavier ni de registres, ces vicissitudes charmantes.
