# Supplementary figures and images for: Using historical accounts of harpsichord touch to empirically investigate the production and perception of dynamics on the 1788 Taskin
Source: Front Psychol. 2015 Mar 11;6:183. doi: 10.3389/fpsyg.2015.00183 (PMC4366653; doi:10.3389/fpsyg.2015.00183)

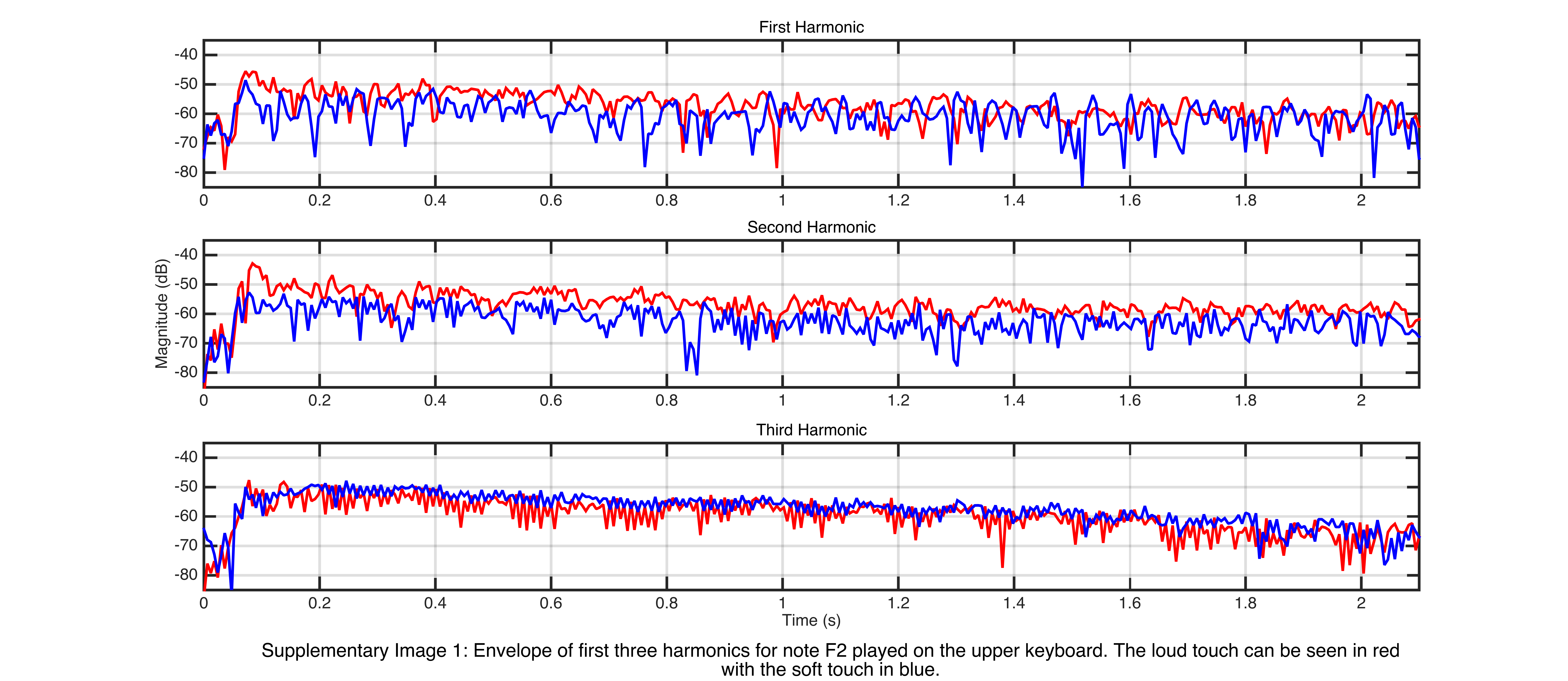

Supplement: Supplementary file 2 [file image_1.tif]

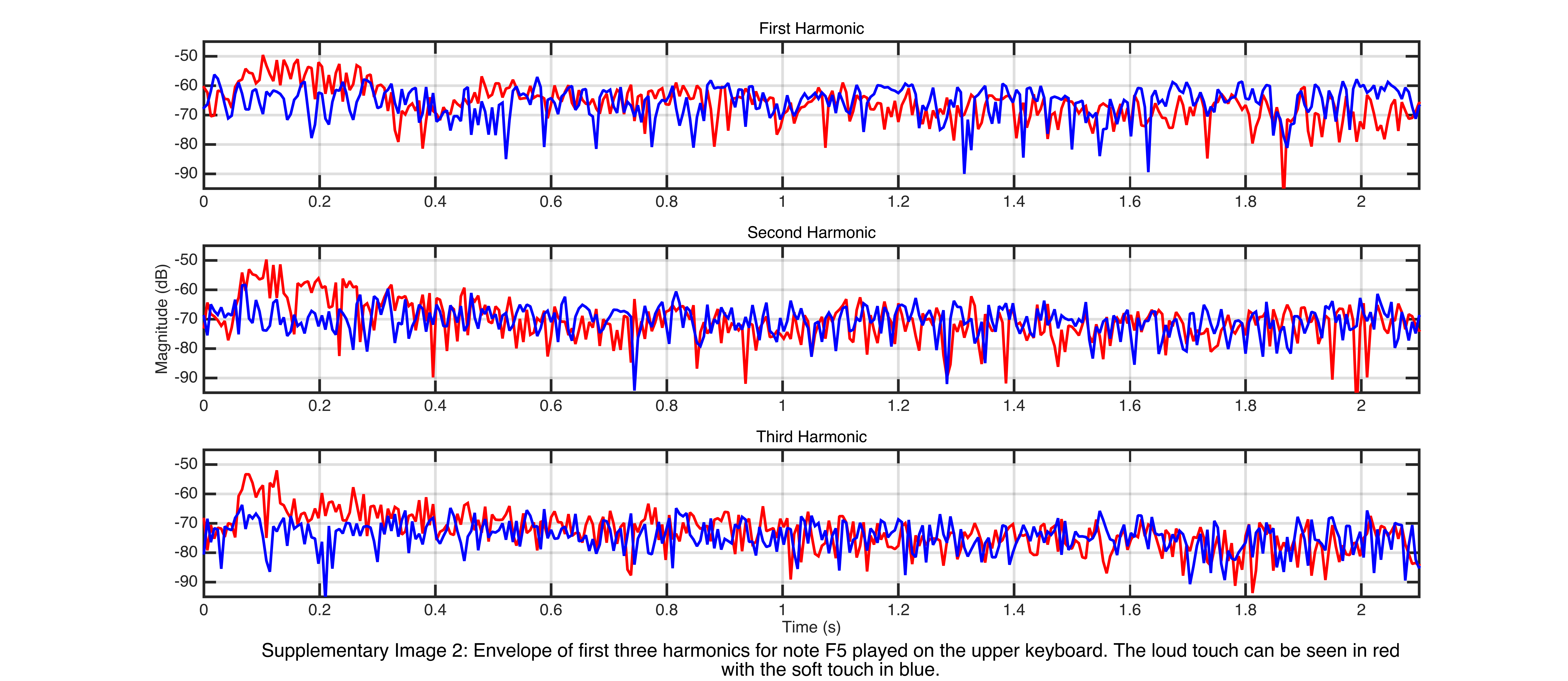

Supplement: Supplementary file 3 [file image_2.tif]

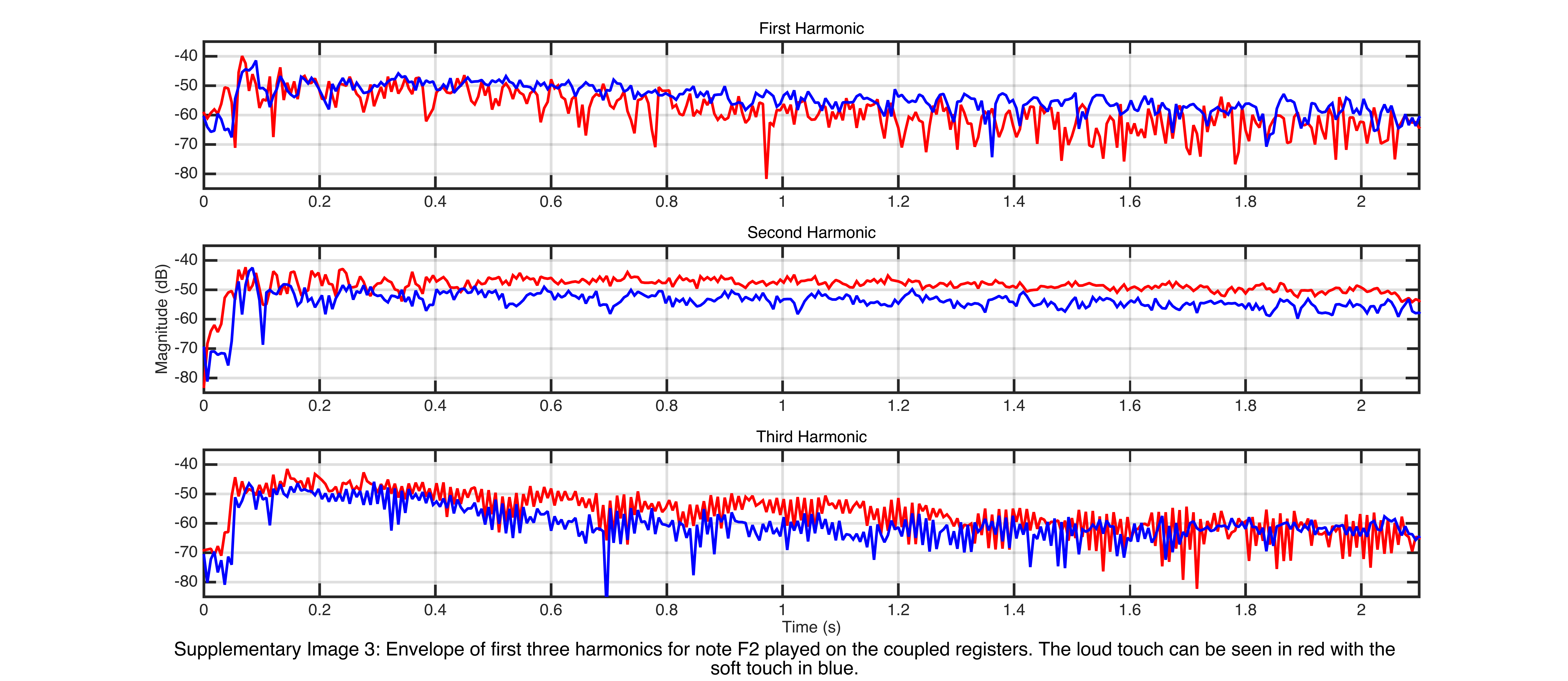

Supplement: Supplementary file 4 [file image_3.tif]

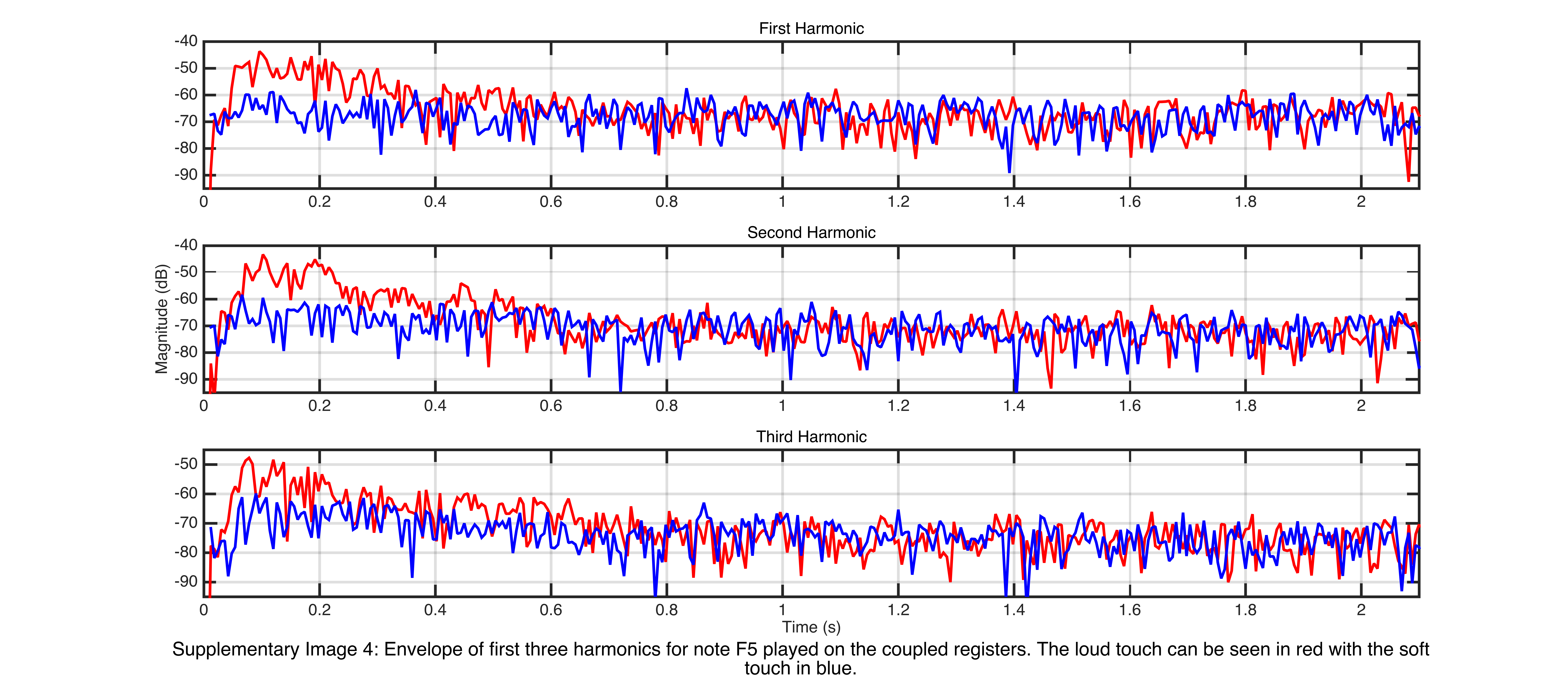

Supplement: Supplementary file 5 [file image_4.tif]
